# Properties of screen printed electrocardiography smartware electrodes investigated in an electro-chemical cell

**DOI:** 10.1186/1475-925X-12-64

**Published:** 2013-07-05

**Authors:** Linda Rattfält, Fredrik Björefors, David Nilsson, Xin Wang, Petronella Norberg, Per Ask

**Affiliations:** 1 Department of Biomedical Engineering, Linköping University, SE-581 83, Linköping, Sweden; 2 Department of Materials Chemistry, Box 538SE-751 21, Uppsala, Sweden; 3 Acreo AB, Sandgatan 31, SE-602 21, Norrköping, Sweden

**Keywords:** Screen printed electrodes, ECG, Electrode impedance, Electrode potential, Smartware electrodes

## Abstract

**Background:**

ECG (Electrocardiogram) measurements in home health care demands new sensor solutions. *In this study, six different configurations of screen printed conductive ink electrodes have been evaluated* with respect to electrode potential variations and electrode impedance.

**Methods:**

The electrode surfaces consisted of a Ag/AgCl-based ink with a conduction line of carbon or Ag-based ink underneath. On top, a lacquer layer was used to define the electrode area and to cover the conduction lines. Measurements were performed under well-defined electro-chemical conditions in a physiologic saline solution.

**Results:**

The results showed that all printed electrodes were stable and have a very small potential drift (less than 3 mV/30 min). The contribution to the total impedance was 2% of the set maximal allowed impedance (maximally 1 kΩ at 50 Hz), assuming common values of input impedance and common mode rejection ratio of a regular amplifier.

**Conclusion:**

Our conclusions are that the tested electrodes show satisfying properties to be used as elements in a skin electrode design that could be suitable for further investigations by applying the electrodes on the skin.

## Background

As a consequence of the increasing number of elderly with cardiovascular diseases there is a healthcare driven need to develop new solutions for home health care. The electrocardiogram (ECG) is an important diagnostic and monitoring modality in point of care systems. This requires a robust wearable ECG acquisition system and smartware has in this context been introduced as a possible solution [1]. Textile electrodes for ECG measurements incorporated in clothes or similar setups have been tested and evaluated [2-5] over the last decade. The textile electrodes, however, often have a complex structure and show e.g. impedance characteristics that depend on for example stretch [4,6]. Printed electronics technology has emerged as a new attractive tool in producing smartware [7,8]. This technology has the potential to provide smartware electrodes with improved characteristics.

The recorded ECG signal consist, besides of the bioelectric activity of the heart, also of contributions from several other sources such as electrode potential drift, liquid junction potentials over the electrolyte and the skin, myoelectric signals and capacitive coupled interferences to the mains [9-11]. If not careful actions are taken to minimize these disturbances, they might seriously distort the ECG. Muscular noise is prevented by suitable location of the electrodes and, if possible, asking the patient to relax. To lower the influence of the other sources, their origins have to be known to be able to reduce their contribution. There are mainly two properties that are of importance, the potential variation and the impedance [9,12].

In this study, we have designed different three layer printed electrodes, using Ag/AgCl- and carbon based inks. The electrodes were tested for electrode potential stability and impedance frequency characteristics under well-defined electro-chemical conditions. Parts of the material have earlier been presented as conference abstracts [13,14].

### Electrode potentials

An electrolyte between the skin and the electrode is necessary to convey the ionic current in the body to the electronic current in the electrode. This electrolyte might be an added electrode gel or sweat accumulated under the electrode. The electrolyte and the electrode hence form a half cell which adds a potential contribution to the ECG measurement.

The half-cell potential, E, for a redox reaction can be calculated from the Nernst equation:

$$E=E_{0}-\frac{\mathrm{RT}}{\mathrm{nF}}\ln\left(\frac{a_{\mathrm{red}}}{a_{\mathrm{ox}}}\right)$$

where *E*_*0*_ is the standard reduction potential for the redox couple, *R* is the gas constant, *T* the temperature, *n* the number of electrons involved in the reaction, *F* the Faraday constant and *a* is the activity of the species. The possible reactions considering measurements on skin, however, are more complex due to the number of involved ions. There is also a liquid junction potential between the electrode gel and the body fluid electrolytes given by a version of the Nernst equation:

$$E_{\mathrm{lj}}=\left(\frac{u^{+}-u^{-}}{u^{+}+u^{-}}\right)\frac{\mathrm{RT}}{\mathrm{nF}}\ln\frac{C_{+}}{C_{-}}$$

Where *u +* and *u-* are the mobilities of the cat- and anions, respectively, and *C*_*i*_ are the electrolyte concentrations. The junction potential is low if the mobility difference between cat- and anions is small and if the mobilities are high [10].

**Figure 1:**
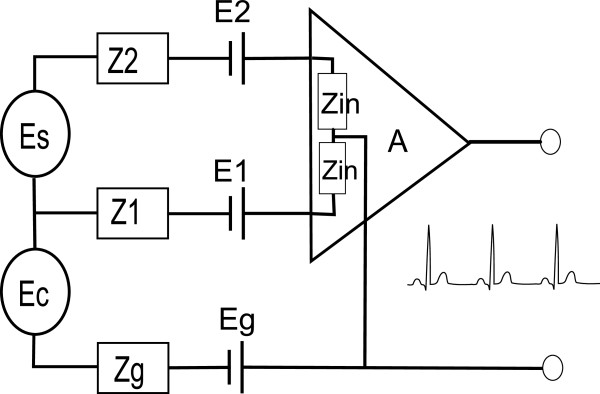
**Simplified description of an ECG measurement setup.** E_s_ is the source signal, E_c_ is the capacitive contribution from the mains and Z_1,2,g_ are the tissue-to-electrode impedances. Z_in_ is the input impedance of the amplifier and A the amplification factor.

When evaluating electrodes in the present experiments it is the open circuit potential, OCP, that is measured (with respect to the reference electrode). The OCP depends on the material of the electrode and electrolyte composition and is the sum of the half-cell potentials and the liquid junction potentials in the system. These potentials are denoted E_1,2,g_ in Figure 1.

Normally when recording an ECG, two or more electrodes are used. Ideally the electrodes and electrolytes are the same at all sites to minimize contributions to the differentially amplified ECG signal. However, if not the same or if the cell is disturbed by movement, a time varying potential difference can occur which is more or less difficult to separate from the signal from the heart. Electrode/electrolyte interfaces need therefore to be tested both for consistency and magnitude of electrode potential. By choosing stable electrode materials and suitable gel electrolytes the time fluctuation of this difference can be minimized. A commonly used clinical setup is a AgCl-based electrode in combination with a Potassium Chloride electrolyte.

### Common mode disturbances

In Figure 1 the ECG measurement setup has been modeled as an electrical circuit where E_s_ is the signal from the heart and Z_in_ is the input impedance of the amplifier. The impedances from the signal sources to the input of the amplifier are denoted *Z*_*1,2,g*_ and are therefore total electrode impedances including both the electrode impedance itself and the skin impedance. Furthermore, the body has a capacitive and inductive coupling to the mains which depends on the presence of electrical equipment, light sources, etc. This coupling acts as a common mode ECG signal disturbance from the power line (50/60 Hz) denoted E_c_ in Figure 1.

The amplifier’s ability to suppress common mode disturbances is expressed as the Common Mode Rejection Ratio, CMRR, defined as:

$$\mathrm{CMRR}=10\log_{10}{\left(\frac{A}{A_{\mathrm{cm}}}\right)}^{2}$$

where *A* is the amplifier’s differential gain and *A*_*cm*_ is the common mode gain. Under the assumption that *Z*_*1*_ and *Z*_*2*_ are much smaller than *Z*_*in*_, the effective common mode rejection ratio is:

$$\mathrm{CMR}R_{\mathrm{tot}}=1/\left(\frac{1}{\mathrm{CMRR}}+\frac{\mathrm{\Delta Z}}{Z_{\mathrm{in}}}\right)$$

Where *CMRR*_*to*t_ is the effective common mode rejection ratio, and ΔZ = *Z*_*2*_**-***Z*_*1*_. This means when looking on a measurement system, the total *CMRR* is not only depending on the CMRR for the amplifier, but also has a component where the impedances *Z*_*1*_ and *Z*_*2*_ are included. In order to have a satisfactory total *CMRR* these impedances should not only be small, but also similar [9].

**Figure 2:**
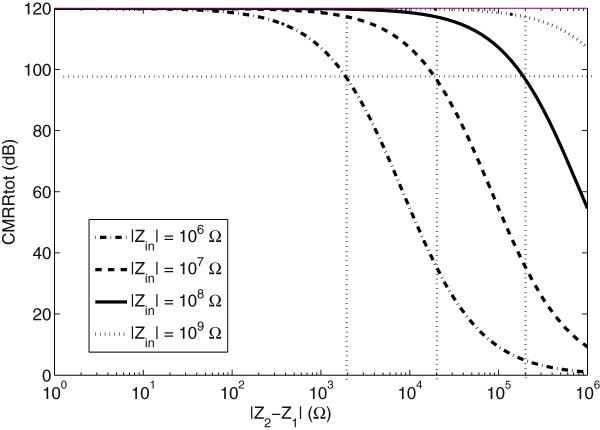
**The effective CMRR as a function of setup parameters.** The CMRR as a function of the total impedance difference between the two electrodes and input impedance in the amplifier.

In Figure 2 the total CMRR is plotted against ΔZ for input impedances of 1, 10, 100 and 1000 MΩ. The amplifier’s CMRR is assumed to be 120 dB [15,16] and a satisfactory limit of noise suppression at line frequency is assumed to be 100 dB [16]. With an input impedance of 10 MΩ and with the assumptions above, a maximum ΔZ of 10 kΩ is obtained. Assuming that the impedance between two sites differs maximally by 20% the highest total impedance magnitude is 50 kΩ. Accordingly, if the input impedance is 100 MΩ, the allowed total impedance is 500 kΩ.

The total skin-electrode impedances in Figure 1 are the impedances from the inside of the body to the input of the amplifier. It can be further decomposed in electrical models for the tissue, skin, electrolyte, electrode and their interfaces, respectively. Included in this setup are also the printed conduction lines. Detailed models can be found in [12,17-19]. Its importance, however, for this study is the proportions between the impedance depending on the outer layer of epidermis; the cornea stratum/electrolyte (here denoted skin impedance) and the impedance of the electrode/electrolyte and the conduction line (here denoted electrode impedance). Normally the skin impedance is much larger than the electrode impedance. However, if the contribution of electrode impedance is large, it will influence *Z*_*1*_, *Z*_*2*_ and hence decrease the *CMRR*.

## Methods

### Screen printed electrodes

**Figure 3:**
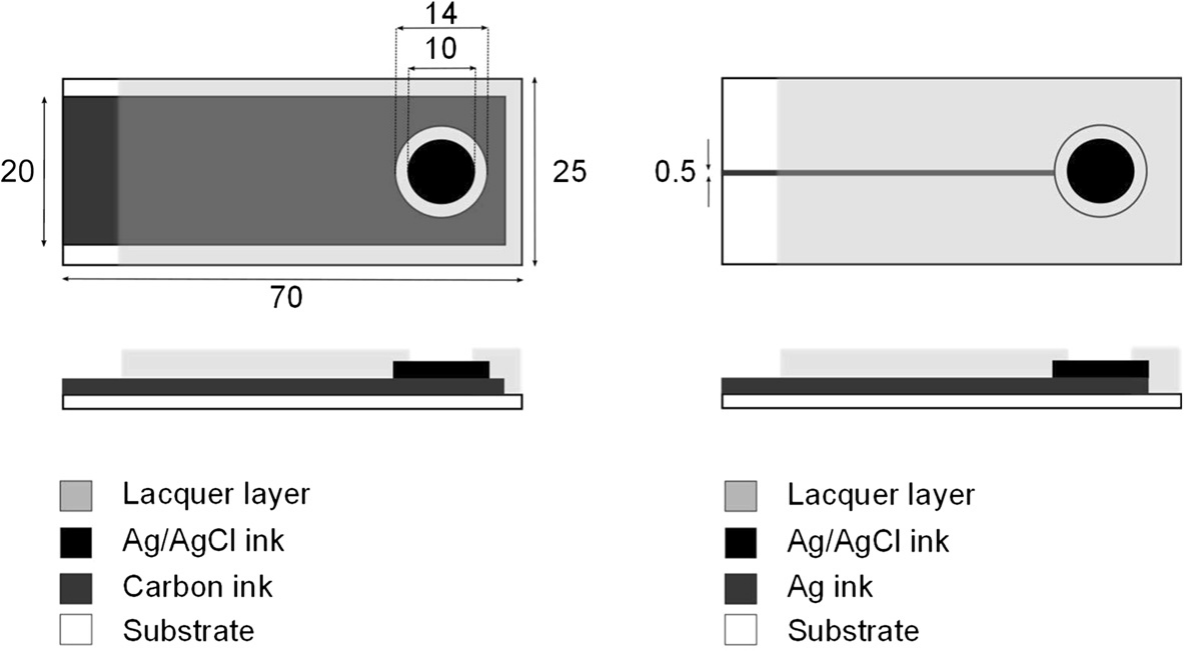
**Schematic drawing of the printed electrodes.** On the left side is the Carbon conductor setup and on the right Ag conductor setup. All measures are in mm.

The electrodes were screen printed on a Polyethylene terephthalate (PET) foil substrate (Polyfoil Bias, 125 Mic). A flat screen printer TIC SCF 550 was used. A first layer of conductive ink was applied as a conduction line. It was either a 20 mm wide carbon based ink (C7102 from Dupont with 10% Dupont 3610 thinner) or a 0.5 mm wide Ag based ink (Ag5000, Dupont), see Figure 3. Curing of carbon and Ag was done with a belt oven for 4 minutes at 140°C. A second layer consisted of a Ag/AgCl ink (Creative Materials, 113–09) circle which served as the electrode area (with a diameter of 14 mm), curing of this layer was done the same way as for carbon and Ag layers. A third layer consisting of insulating lacquer (SericolUvivid Screen CN-622 Tactile Varnish) was applied resulting in an effective electrode diameter of 10 mm, curing of the lacquer layer was done with an Aktiprint UV dryer. In order to investigate the importance of layer thickness, a second layer of Ag/AgCl and lacquer were reprinted in a number of the samples. Each type of electrode was printed simultaneously. The different specimens are summarized in Table 1. For comparison, measurements were also made on commercially available skin electrodes, denoted as ‘commercial electrodes’ (Suction Electrode KISS, GE Healthcare, 20 mm in diameter).

**Table 1:** Tested electrode configurations

**Denotation**	**Conduction line material**	**Layers of Ag/AgCl**	**Layers of lacquer**	**No of samples**
A_Ag_	Ag	1	1	8
A_C_	C	1	1	7
B_Ag_	Ag	1	2	8
B_C_	C	1	2	8
C_Ag_	Ag	2	1	8
D_Ag_	Ag	2	2	6
Commercial	-	-	-	4

### Measurement setup

The open circuit potential, OCP, and impedance for the electrodes were evaluated in an electrochemical cell consisting of physiological saline solution (0.9% NaCl). The electrodes were mounted on a rigid fixture and submerged in the electrolyte. A potentiostat, Autolab PGSTAT30 (MetrohmAutolab, Utrecht) was used for the measurements along with a Ag/AgCl reference electrode and a palladium auxiliary electrode. The open circuit potential was measured with one minute intervals during 30 minutes.

Impedance measurements were performed in the range 0.05 Hz to 2 KHz (50 samples, logarithmically distributed) with a root mean square amplitude of 10 mV at the open circuit potential.

### Data processing

For the electrode potential measurements, the mean drift/time, ${\bar{E}}_{i}$, was calculated for each electrode type, *X* = A_Ag_, A_C_,…. as the mean absolute difference between two consecutive samples:

$$\begin{array}{l} {\bar{E}}_{X,i}=\frac{\sum {}_{t}\left|E_{X,i}\left(t\right)-E_{X,i}\left(t+1\right)\right|}{29},t=1...29\\ {\bar{E}}_{X}=\frac{\sum {}_{i}E_{X,i}}{n},i=1...n \end{array}$$

Where *i* denotes the specimen and *n* the number of specimens of electrode type *X*. Since a few outliers were present in the dataset, the medians were chosen to present each electrode type in the electrode impedance plots. Impedance values at 1, 15, 50 and 2000 Hz (mean, confidence interval) were used for evaluating the impedance properties.

## Results

### Electrode potential

**Figure 4:**
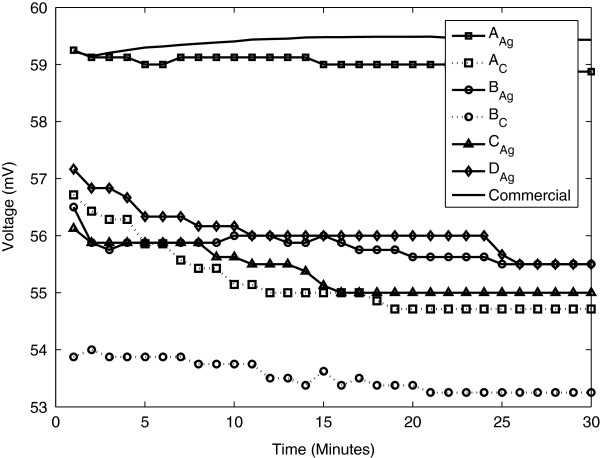
**Mean electrode potential for each electrode.** The commercial electrode has the highest potential followed by A_Ag_.

In Figure 4 the mean electrode potential is displayed for the six electrode configurations and the commercial ones. As can be seen, it varies between 53 and 59 mV. The electrode potential within the same electrode type is consistent both over time and between specimens. The largest difference between samples in one batch was for the B_C_ electrode with a maximum difference of 5 mV. For the other electrode types the corresponding values were approximately 2–3 mV. The standard deviation between specimens of the same type at a given time was 0.5 to 0.8 mV, electrode B_C_ having a slightly higher value of 2 mV (commercial electrode 0.3 mV). The decrease from start to end is lower than 3 mV for all individual electrodes. The mean potential drift is in the range of 0.03 mV/min (A_Ag_) to 0.08 mV/min (B_C_). Corresponding value for the commercial electrode is 0.02 mV/min.

### Electrode impedance

**Figure 5:**
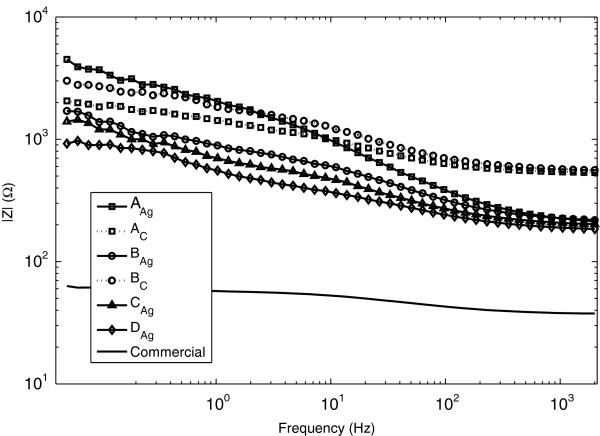
**Absolute value of the impedance for each electrode type.** Median values are used.

The median absolute values of the impedances at different frequencies are shown in Figure 5. It can be seen that at 2 kHz the Carbon conductor electrodes have a higher resistance than the Ag-based conductor lines. For the higher frequencies, the electrodes with Ag conduction lines behave similarly, but with decreasing frequency, electrode type A_Ag_ has much higher impedance.

**Figure 6:**
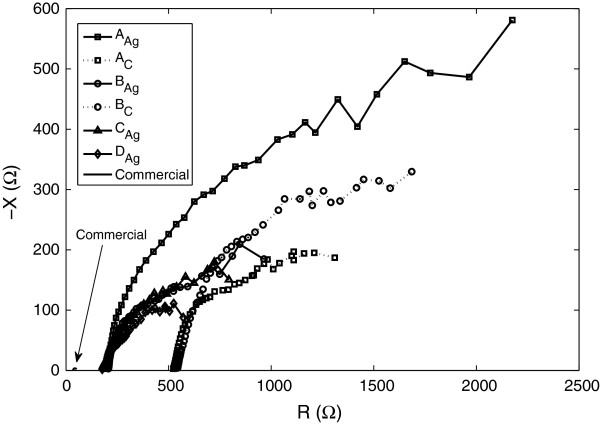
**Impedance plot of all electrode types.** Median values are used.

In Figure 6 the measurement results are represented in an impedance plot. It can be seen that the reactance is capacitive but that the dominant part of the impedance is resistive.

In Table 2, the mean and 95% confidence intervals of the impedances are specified for the different electrode types for frequencies corresponding approximately to the heart rate, QRS-complex, line frequency and maximum acquisition frequency. For noise suppression, especially the 50 Hz frequency is of interest. The confidence intervals are larger for low frequencies since they are more prone to be influenced by background noise. The maximal impedance at 50 Hz is of magnitude 1 kΩ.

**Table 2:** Impedances at some frequencies for the electrodes

**Electrode**	**|Z| at 1 Hz**	**|Z| at 15 Hz**	**|Z| at 50 Hz**	**|Z| at 2000 Hz**
A_Ag_	4200 (2200–6100)	1600 (1100–2200)	820 (600–1000)	240 (210–260)
A_C_	2200 (1200–3200)	1200 (1000–1300)	820 (800–850)	570 (520–620)
B_Ag_	1880 (860–2800)	880 (760–1000)	560 (470–660)	260 (210–310)
B_C_	3400 (1900–5000)	1600 (1400–1700)	1000 (900–1100)	580 (530–630)
C_Ag_	1300 (600–2100)	660 (490–830)	440 (350–520)	230 (190–270)
D_Ag_	750 (530–980)	440 (340–550)	340 (260–420)	200 (170–230 )
Commercial	57 (47–68)	52 (43–60)	46 (38–53)	38 (32–43)

The values are the mean and the 95% confidence interval (in brackets).

## Discussion

In this study, screen printed electrodes made of conductive ink have been tested for electrode potential stability and electrode impedances.

Electrode potential drift can cause a disturbance in the measured signal which is much larger than the ECG amplitude (approx. 1 mV). In order to estimate the possible effect the drift might have on the measured signal, consider the relatively small P-wave of the ECG. Assuming an amplitude of 0.3 mV and a duration about 120 ms [11] gives a slope of 2.5 mV/s. Scaling the electrode potential drift correspondingly, its maximum slope was 0.08 mV/min corresponding to 0.0013 mV/s. There is a factor 10^3^ between the slopes which seems to be sufficient for the drift not to inflict on the ECG signal. The obtained results therefore indicate that the stability is adequate for the different electrode configurations. The potentials were also very consistent within each electrode type both regarding over time and between specimens. The printed electrodes in this sense performed comparable with the commercially available electrodes.

The magnitude of the electrolyte-electrode impedance at 50 Hz is an important parameter when it comes to noise suppression of artifacts induced by the mains. Here, two factors are of importance, the ratio between the skin impedance and the electrode impedance and the sum of these in comparison to the total input impedance of the amplifier. With the assumptions made in the introduction, the maximum allowed impedance difference between the two electrode sites should be 50 kΩ. The maximal mean value of electrode impedance measured at 50 Hz was 1 kΩ (B_C_) and hence is a factor 50 less than the allowed limits. It is potentially valid for only 2% of the impedance difference and should hence not be considered as a limiting factor of the entire system.

High frequency (2 kHz) measurements show that the mean Silver conduction line electrodes have an impedance of approximately 200–250 Ω while the Carbon conduction line electrodes have corresponding value of 570–580 Ω. We believe that this discrepancy (320–380 Ω) is due to the difference of conductance in the conduction lines. While looking at the total measurement system, however, also the conduction lines will be added to the impedance which is coupled to the amplifier and is hence not negligible.

In this experimental setup, the skin-electrode interface has not been included. Extensive work in this area has been performed elsewhere for living tissue [20] and tissue models [17,21] experiments. For the aim of this study it is sufficient to acknowledge that there can be large differences in skin impedances. For example by using regular tape to strip the skin, the impedance was reduced from 200 kΩ to 1 kΩ at 1 Hz after 9 repetitions [20]. For the application in mind, the electrodes should be used on unprepared skin. However, differences in skin impedance can be large depending on the humidity and local properties of the skin. For example, the thickness of the stratum cornea varies with for example gender, age, location and humidity [22]. The skin impedance also differs much depending on frequency. Rosell et al. [23] for example, stated that the skin impedance varies from around 200 kΩ at 1 Hz to 200 Ω at 1 MHz. A commonly used value for impedance for unprepared skin without gel is in the 100 kΩ range [2,19,24]. This would imply that the input impedance of the amplifier rather should be in the 100 MΩ range to provide sufficiently CMRR for the entire system. The impedance contribution from the electrodes themselves is, however, small.

There is a standard for disposable ECG electrodes published by the Association for the Advancement of Medical Instrument (ANSI/AAMI EC12:2000). As described by Xu et al. [21] it states that between two electrodes connected gel-to-gel, the maximal allowed impedance is 2 kΩ at 10 Hz (with a current less than 100 microA). Furthermore, the DC offset after one minute of stabilization is to be less than 100 mV using the same setup. The standard is only valid for pre-gelled disposable ECG electrodes. The tested electrodes here are without gel or paste and hence cannot be compared according to the standard. Instead the measurements were performed in a controlled electro-chemical environment.

In order to improve the systems’ CMRR, the amplifiers’ characteristics can be improved. A general background on differential amplifiers’ CMRR is given in [25]. Implementations with high input impedance are described in [24] and a solution with pre-amplified electrodes is given in [26].

By using a smooth plastic film in the electrodes, the intention was to decrease the influence of the substrate and increase the impact of the print itself in the measurements. When using textiles, the properties of the electrodes are not only defined by the ink but also vary quite drastically due to the combination of textile and printing method. Two common modalities of measuring electrical properties of prints are sheet resistance and time domain reflectometry. Such investigations have been performed on prints on fabric by [8,27,28] (sheet resistance) and [28,29] (time domain reflectometry). However, in the application of electrodes, the print is in contact with an electrolyte which adds a frequency dependency. The methodology used in this study therefore includes submerged electrodes.

For the final application of having printed electrodes included in garments, several other factors need to be addressed; the print could be made on textiles and conduction lines should be integrated in the garment. Furthermore, the garment might need to be washable. Kazani et al. [8] have used textile substrates for conductive ink screen printing and investigated its electrical properties before and after washing the fabric. It was concluded that some of the inks and substrates together with a coating still remained highly conductible even after 20 washing cycles.

## Conclusion

The conclusion that can be drawn from the present experiments is that this particular ink, the Ag/AgCl, has good properties regarding electrode stability when printed on a plastic foil substrate. All electrode configurations show reasonable electrode impedances which, included in an appropriate measurement setup with high input impedance amplifiers, should not be a limiting factor. Future studies with prints on textiles and measurements on skin are, however, necessary in order to decide its applicability in tomorrow’s sensor technology.

## Competing interests

The authors certify that they have no competing interests.

## Author´s contributions

LR has been involved in the study design, made the data acquisitions and made the main part of the drafting of the manuscript. FB has substantially contributed with the methodology concerning electro-chemical measurements. DN, XW and PN have contributed in the design and conception of the printed electrodes. PA made the overall study design and contributed to the manuscript. All authors have revised the manuscript and have approved the final manuscript.
